# Exploring the antimicrobial activity of fermented and non‐fermented cocoa bean shell extracts through metabolomics analysis and synergistic studies

**DOI:** 10.1002/jsfa.14366

**Published:** 2025-05-13

**Authors:** Yie Kie Chong, Wei Khang Gan, Joash Ban Lee Tan, Ahmad Kamil Hj Mohd Jaaffar, Zainal Baharum, Keng Yoon Yeong

**Affiliations:** ^1^ School of Science Monash University Malaysia Campus Bandar Sunway Malaysia; ^2^ Malaysian Cocoa Board Kota Kinabalu Malaysia

**Keywords:** antimicrobial, cocoa bean shell, fermentation, metabolomics, synergy

## Abstract

**BACKGROUND:**

Cocoa bean shell (CBS) extracts have emerged as a promising source of antimicrobial compounds. However, the bioactive compounds responsible for their antimicrobial activity have not been studied sufficiently. This study analyzed the antimicrobial properties of 12 extracts from fermented and non‐fermented CBS, employing solvent and steam distillation extraction techniques to maximize bioactive diversity. The extracts were assessed against selected bacteria and fungi, including the ‘ESKAPE’ pathogens.

**RESULTS:**

The CBS solvent and steam distillation extracts (except for ethyl acetate‐fermented CBS) were found to exhibit antimicrobial activity against *Streptococcus mutans* with minimum inhibitory concentrations (MICs) as low as 0.0625 mg mL^−1^, whereas both fermented and non‐fermented steam distillation CBS extracts were found to be active against *Candida albicans* with MICs at 1 mg mL^−1^. Overall, steam distillation extracts possessed enhanced antimicrobial activity in comparison with solvent extracts of CBS. Fermented CBS extracts were found to possess better antimicrobial activity than non‐fermented CBS extracts. Metabolomics analysis identified theobromine (TB) and tetramethylpyrazine (TMP) as molecules that contributed to antimicrobial activity against *S*. *mutans*. Results showed that caffeine (CAF), TB and TMP were active against *S. mutans* and *Acinetobacter baumannii*, whereas CAF and TB were active against *C*. *albicans*. Significant synergistic effects of CAF, TB, and TMP with ciprofloxacin (CIP) were observed against *Klebsiella aerogenes.*

**CONCLUSION:**

These findings highlight the significant potential of bioactive compounds present in CBS for use in the development of sustainable antimicrobial agents. These naturally occurring compounds, including CAF, TB, and TMP, exhibited notable antimicrobial properties, which could also be harnessed to enhance the activity of commonly used antibiotics such as ciprofloxacin. Future studies could focus on determining the mode of action of these bioactive molecules. © 2025 The Author(s). *Journal of the Science of Food and Agriculture* published by John Wiley & Sons Ltd on behalf of Society of Chemical Industry.

## INTRODUCTION

Antimicrobial multidrug resistance (AMR) is a pressing global health issue exacerbated by the overuse and misuse of antibiotics across various sectors, including medicine, agriculture, livestock, and aquaculture.^1^ Although the development of new antibiotics is crucial for combating AMR, it has become increasingly difficult to bring new antibiotics to market, mainly due to the high costs involved and the exit of major pharmaceutical companies from this area.

Driven by the idea to lower the cost of antibiotic drug discovery as well as sustainable management of food and agricultural waste, the waste from natural products has often been explored for its antimicrobial potential. It has been shown repeatedly to offer a promising alternative to conventional drug discovery by harnessing the chemical diversity found in these underutilized resources.^2^, ^3^, ^4^, ^5^, ^6^, ^7^

**Figure 1:**
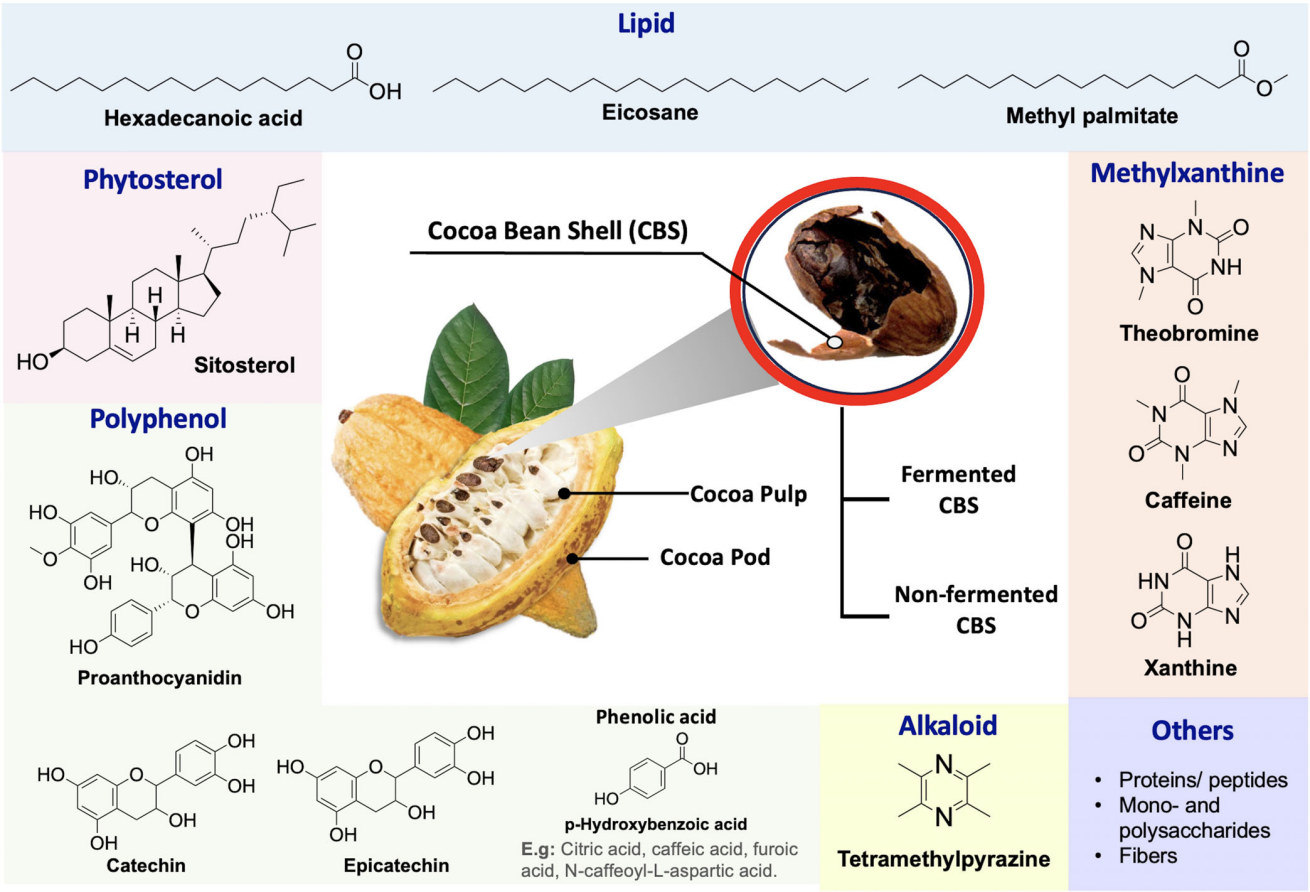
Bioactive compounds that commonly present in the cocoa bean shell (CBS).

Malaysia is one of the largest cocoa traders in Asia and it generates substantial cocoa waste during industrial processing.^8^ Cocoa waste poses environmental challenges, leading researchers to explore ways to repurpose it for beneficial uses. One of the avenues is to explore its medicinal potential as this waste is known to be rich in bioactive compounds such as polyphenols, methylxanthines, and lipids (Fig. 1). Cocoa bean shell (CBS) is one of the main by‐products. It represents approximately 10% to 17% of the total bean weight and is removed from the cotyledons before or after the roasting process.^9^ Preliminary reports in recent years have highlighted the antidiabetic,^10^ antioxidant,^11^ antimicrobial,^12^, ^13^, ^14^, ^15^ and anti‐inflammatory^16^, ^17^, ^18^ properties of CBS. The positive findings regarding its antimicrobial activity prompted this investigation into its potential.

Cocoa bean fermentation is a common step in cocoa processing. This process affects the phenolic content, protein, and fat content of cocoa.^19^, ^20^, ^21^ As far as the authors of the current study are aware, studies on the antimicrobial activity comparison of fermented and non‐fermented CBS extracts have yet to be reported.

This study analyzed the bioactive compounds that are present in fermented and non‐fermented CBS using various extraction protocols. The antimicrobial activities of these extracts were evaluated against gram‐positive and gram‐negative bacteria including the ‘ESKAPE’ pathogens, and *Candida albicans*. Each CBS extract exhibited varying bioactive compound content. Metabolomics analysis was also performed to investigate the relationship between the bioactive profiles of CBS extracts and their antimicrobial ability, as well as to identify the key compounds associated with their inhibitory activity. Selected CBS bioactive compounds were further evaluated for their antimicrobial activity and synergistic effect with ciprofloxacin.

## MATERIALS AND METHODS

The fermented and non‐fermented dried CBS were obtained from the Malaysian Cocoa Board (Nilai, Negeri Sembilan, Malaysia). The fermented and non‐fermented CBS were prepared following a method described in the literature.^22^, ^23^ Briefly, fresh cocoa beans (100 g) were fermented at room temperature for 5 days, followed by a second 5‐day fermentation with the addition of distilled water (60 mL). De‐shelling was performed via infrared micronization at 130 °C for 2 min at 600 rpm. The beans were then broken using a bean breaker, and the shells were separated from the nibs with a winnower. Non‐fermented CBS were de‐shelled using the same protocol, omitting the fermentation steps.

### Solvent extraction of fermented CBS and non‐fermented CBS

The extraction of CBS was performed according to previous reports with minor modifications.^24^, ^25^ The samples were defatted before the extraction procedure. Briefly, ground CBS (50 g) was added to hexane (250 mL) and the mixture was stirred for 5 min. After stirring, the mixture was vacuum filtered and the process was repeated twice. The defatted samples were dried in an oven at 50 °C for 4 h. Five different solvents were used: ethyl acetate, ethanol, methanol, acidified ethanol‐water (pH 3, 80:20 v/v), and acidified methanol–water (pH 3, 80:20 v/v). The pH was adjusted using 0.1 mol L^−1^ hydrochloric acid. Solvent extraction was carried out using a reflux system. Cocoa bean shell (5 g) was added to each extraction solvent (15 mL) and refluxed for 60 min with stirring. Each solvent was heated to its boiling point or slightly above: ethyl acetate and ethanol at 78 °C, methanol at 65 °C, acidified ethanol‐water at 80 °C, and acidified methanol–water at 70 °C. After it cooled to room temperature the filtrate was collected. The extraction process was repeated twice, and the collected filtrates were then combined and subsequently dried *in vacuo* to give the final extracts.

### Steam distillation

Cocoa bean shell (60 g) and distilled water (600 mL) were mixed in a round‐bottom flask. The mixture was heated to boil on a heating mantle for 1.5 h. Around 100 mL of distillate was collected into the receiving flask. After the distillate was extracted with dichloromethane (DCM) (150 mL × 3), the organic solvent was removed *in vacuo* to obtain the steam distilled extract fraction.

### Liquid chromatography–quadrupole time‐of‐flight mass spectrometry

The liquid chromatography–quadrupole time‐of‐flight mass spectrometry (LC‐QTOF‐MS) data were captured using an Agilent 1290 Infinity LC system coupled with the Agilent 6520 Accurate‐Mass quadrupole time of flight (QTOF) mass spectrometer (Agilent, Santa Clara, CA, USA). The chromatographic separation was achieved using an Agilent Eclipse XDB‐C18 column (150 mm × 2.1 mm, Agilent). The mobile phase consisted of 0.1% formic acid in water: 0.1% formic acid in acetonitrile, applied in a gradient from 95:5 (0–5 min) to 0:100 (5–20 min), followed by isocratic elution at 0:100 (20–25 min), at a flow rate of 0.5 mL min^−1^. Data were acquired using Agilent MassHunter qualitative analysis B.07.00 software. Both positive and negative ionization modes were used. Secondary metabolites were identified by comparison with the METLIN database; compounds with a matching score below 80% were excluded from further analysis.

### Gas chromatography–mass spectrometry

A QP2010 Ultra (Shimadzu, Kyoto, Japan) gas chromatography–mass spectrometry system was fitted with a Zebron ZB‐5MS column (30 m × 0.25 mm × 0.25 μm) of film thickness and maximum temperature of 350 °C. Ultra‐high‐purity helium (99.99%) was used as a carrier gas at a constant flow rate of 1.0 mL/min. The injection, transfer line, and ion source temperatures were all set at 280 °C. The oven temperature was programmed to start at 80 °C (for 2 min) and increased to 280 °C at a rate of 3° C/min. Crude extracts were diluted with a suitable solvent (1/100, v/v), filtered, and 1 μL of the diluted extract was injected with a split ratio of 10:1. Full‐scan mass spectra were collected over a scan range of 40–550 amu. The percentage composition of the crude extract constituents was determined by the percentage of peak area, with chemical compounds identified based on gas chromatography (GC) retention time and matched against the NIST 08 mass spectral library.

### Statistical analyses

Statistical analyses of the untargeted metabolomic studies were conducted using the one factor module in MetaboAnalyst 6.0.^26^ The dataset was sum normalized, log transformed, and Pareto scaled to assess the variance in antimicrobial activity against *Streptococcus mutans* (active, moderately active, less active, and inactive) in fermented and non‐fermented CBS extracts using principal component analysis (PCA) and partial least‐squares discriminant analysis (PLS‐DA). Principal component analysis, an unsupervised multivariate analysis, used five components to visualize sample clustering based on metabolic profiles. Partial least‐squares discriminant analysis, a supervised method, was used to identify significant differences in metabolites extracted from fermented and non‐fermented CBS extracts. Model validation by permutation testing yielded *P* < 0.01, and fivefold cross‐validation was used to estimate prediction error and calculate *Q* ^2^ and *R* ^2^ values for the five components. Principal component analysis and PLS‐DA were not performed on steam‐distilled CBS extracts (F‐SD and NF‐SD) due to limited data. Variable importance in projection (VIP) scores were calculated to identify metabolites contributing to separation in the PLS‐DA plot; VIP scores greater than 1 indicated metabolites strongly discriminating between active and inactive samples. A heatmap was also generated to visualize metabolite concentrations across CBS extracts.

### General protocol for antimicrobial activity test

#### Broth microdilution assay

The broth microdilution assay was conducted according to the Clinical and Laboratory Standards Institute (CLSI) guidelines.^27^ All bacteria strains were cultured on Mueller–Hinton agar (MHA), except for *S. mutans*, which was cultured on trypticase soy agar (TSA), and *C. albicans*, which was cultured on potato dextrose agar. After 24 h of incubation, the colonies were transferred into their respective broths and incubated for a further 24 h at 37 °C. The resulting turbid suspensions were then adjusted to a 0.5 McFarland standard containing 1 × 10^6^ CFU mL^−1^. After inoculation, the 96‐well plates were incubated at 37 °C for 18 h. Minimum inhibitory concentrations (MICs) were determined visually as the lowest concentration at which no visible growth was observed. Ciprofloxacin (CIP) and cycloheximide served as positive controls for bacterial strains and *C. albicans*, respectively. All experiments were performed in triplicate.

### Synergistic study of the CIP‐phenolic compound from CBS waste

The synergistic effects between CIP and compounds derived from CBS waste were evaluated against a range of microorganisms, including methicillin‐resistant *Staphylococcus aureus* (MRSA) ATCC 33591; methicillin‐susceptible *S. aureus* (MSSA) ATCC 25923 and ATCC 6538; *Klebsiella pneumoniae* ATCC 10031; *Enterococcus faecalis* ATCC 29212; *E. faecium* ATCC 700221; *Bacillus cereus* ATCC 14579; *Pseudomonas aeruginosa* ATCC 10145; *Acinetobacter baumannii* ATCC BAA‐1605 (multidrug resistant); *A. baumannii* strains C28, C65, and C98 (isolated from Segamat Hospital, Johor, Malaysia);^28^ *Escherichia coli* ATCC 10798; *S. mutans* ATCC 25175; *B. subtilis* ATCC 8188; *K. aerogenes* ATCC 13048; and *C. albicans* ATCC 10231.

The synergistic effects of CIP combined with bioactive compounds theobromine (TB), caffeine (CAF), and tetramethylpyrazine (TMP) present in the CBS were evaluated using the checkerboard dilution method with minor modifications.^29^ All compounds were tested for their synergistic effects at their maximum concentration in different solvents. Theobromine was dissolved in 0.5 mol L^−1^ of NaOH water, CAF was dissolved in Mueller–Hinton broth (MHB), and TMP was dissolved in methanol. Each solvent was tested against the corresponding bacteria and fungi to ensure that it did not contribute to the killing at its final concentration. Serial dilutions of CBS compounds in combination with CIP were mixed in MHB. The inoculations were prepared from colonies grown overnight on MHA, except for *S. mutans*, which was grown in trypticase soy agar (TSA) and *C. albicans*, which was grown in potato dextrose agar (PDA). The synergistic effect was determined after incubation at 37 °C for 18 h. Each experiment was replicated three times. The *in vitro* interaction between the CBS compounds and CIP was quantified by determining the fractional inhibitory concentration index (FICI) using Eqn (1). The FICI of each compound was interpreted as follows: FICI of 0.5 or less indicates synergy; FICI between 0.5 and 1 indicates additivity; FICI between 1 and 2 indicates no effect, and FICI greater than 2 indicates antagonism.^30^

(1) $$\text{FICI value}=\text{FICI}\;\left(X\right)+\text{FICI}\;\left(\mathrm{CIP}\right)=\frac{\left[X\right]}{X\;\mathrm{MIC}}+\frac{\left[\mathrm{CIP}\right]}{\mathrm{CIP}\;\mathrm{MIC}}$$

where *X* = concentration of TB, CAF, or TMP.

## RESULTS AND DISCUSSION

This study investigated the phytochemical composition and antimicrobial activity of fermented and non‐fermented cocoa bean shell (CBS) extracts. Twelve fermented (F) and non‐fermented (NF) CBS extracts were yielded from solvent extraction using ethyl acetate (F‐EtOAc, NF‐EtOAc), acidified ethanol (F‐A‐EtOH, NF‐A‐EtOH), ethanol (F‐EtOH, NF‐EtOH), acidified methanol (F‐A‐MeOH, NF‐A‐MeOH), methanol (F‐MeOH, NF‐MeOH), and steam distillation (F‐SD, NF‐SD). The non‐volatile phytochemical content of solvent extracts was determined by LC‐QTOF‐MS and the volatile content from steam distillation was analyzed using gas chromatography–mass spectrometry (GC–MS).

### Analysis of the Non‐volatile and Volatile Phytochemicals from Fermented and Non‐fermented CBS Extracts

**Figure 2:**
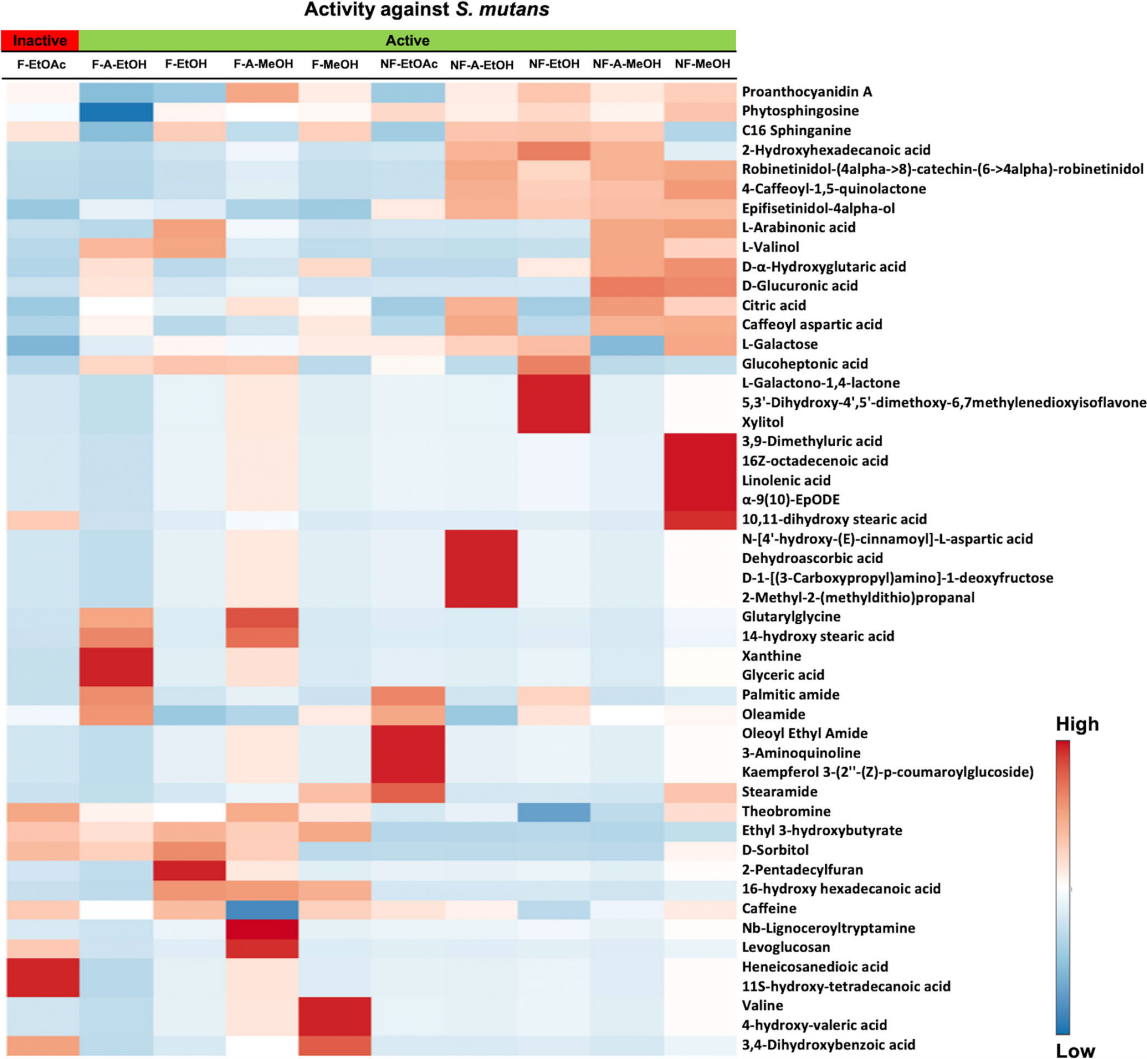
Heatmap generated by MetaboAnalyst software, represents the abundance of metabolites from both fermented (F‐EtOAc, F‐A‐EtOH, F‐EtOH, F‐A‐MeOH and F‐MeOH) and non‐fermented CBS extracts (NF‐EtOAc, NF‐A‐EtOH, NF‐EtOH, NF‐A‐MeOH and NF‐MeOH). The color scale ranged from red (higher abundance) to blue (lower abundance).

A total of 50 compounds were proposed from the LC‐QTOF‐MS analysis of both fermented and non‐fermented CBS solvent extracts. The non‐volatile compounds and their volume peak intensity of fermented extracts (F‐EtOAc, F‐A‐EtOH, F‐EtOH, F‐A‐MeOH and F‐MeOH) and non‐fermented extracts (NF‐EtOAc, NF‐A‐EtOH, NF‐EtOH, NF‐A‐MeOH and NF‐MeOH) are summarized in Supporting Information, Table S1, and depicted in Fig. S1, and the metabolites in each extract are illustrated in the heatmap in Fig. 2. In general, the major compounds that were found in fermented and non‐fermented solvent extracts are methylxanthines (TB, CAF) and polyphenol (proanthocyanidine, catechin derivatives, and caffeoyl derivatives) and a large variety of lipids (sphinganine, oleamide, and *N*‐pentadecylcyclohexanecarboxamide), with non‐fermented extracts exhibiting more diversity than fermented CBS. Higher concentrations of TB and CAF were found in fermented CBS extracts. This finding is in line with the results, where the non‐fermented CBS exhibited less TB and CAF content due to exudation from the bean to the shell after fermentation.^19^, ^31^, ^32^, ^33^, ^34^

Forty‐six volatile compounds were detected from the GC–MS analysis of fermented (F‐SD) and non‐fermented (NF‐SD) CBS steam distillation extracts. Most detected compounds are lipids, with the non‐fermented extracts containing a greater variety of lipids than fermented CBS. Supporting Information, Table S2 contains a list of volatile compounds found in F‐SD and NF‐SD. They are depicted in Supporting Information, Fig. S2. It is notable that TMP was found only in fermented CBS (F‐SD).

This study provided valuable insights into the phytochemical composition of fermented and non‐fermented CBS identified with LC‐QTOF‐MS and GC–MS analyses. However, the absence of structural confirmation from nuclear magnetic resonance (NMR) spectroscopy or further analytical data limited a more conclusive analysis of the compounds.

### Antimicrobial Activity of Fermented and Non‐fermented CBS Extracts

The antimicrobial activity of the extracts was assessed against a panel of bacteria and fungus as outlined in Supporting Information, Table S3. The results revealed that none of the extracts exhibited bacterial inhibition at concentrations of 2 mg mL^−1^ or lower, except for *S. mutans* and *C. albicans* (Table 1). Apart from F‐EtOAc, all CBS extracts demonstrated inhibition against *S. mutans*, with MICs ranging from 0.0625 to 2 mg mL^−1^. Both fermented and non‐fermented steam distillation extracts displayed significant inhibition against *C. albicans*, with MICs of 1 mg mL^−1^. Several studies have investigated the antimicrobial activity of extracts derived from cocoa bean, cocoa pod husk and CBS, with most of the cases reporting higher MIC values. It was reported that the cocoa powder extract exhibited MIC of 5 mg/mL against *C. albicans, E. coli, S. aureus*, and *P. aeruginosa*, while *C. albicans* was the most sensitive microorganism with the lowest MIC of 5 mg mL^−1^.^35^ They also found that the cocoa powder extract had slightly higher potential against gram‐positive bacteria than gram‐negative bacteria. One study found that crude cocoa husk water–methanol extract inhibited *S. choleraesuis* and *S. epidermidis* with minimum inhibitory concentrations (MICs) of 1 mg mL^−1^ and 2.5 mg mL^−1^, respectively.^36^ Another study similarly demonstrated antimicrobial activity of a CBS ethanol–water extract against *S. mutans*, with an MIC as low as 0.0625 μg·mL^−1^. However, no activity was observed against MSSA, MRSA, *E. coli*, *P. aeruginosa*, *C. albicans*, or *S. cerevisiae*.^12^

**Table 1:** The minimum inhibitory concentrations (MICs) of fermented and non‐fermented cocoa bean shell (CBS) extracts

Sample ID	Minimum inhibitory concentrations (MIC) (mg mL^−1^)	
	*S. mutans* (ATCC 25175)	*C. albicans* (ATCC 10231)
**F‐EtOAc**	>2	>2
**F‐A‐EtOH**	2	>2
**F‐EtOH**	0.5	>2
**F‐A‐MeOH**	0.5	>2
**F‐MeOH**	0.5	>2
**F‐SD**	0.0625	1
**NF‐EtOAc**	1	>2
**NF‐A‐EtOH**	1	>2
**NF‐EtOH**	2	>2
**NF‐A‐MeOH**	1	>2
**NF‐MeOH**	1	>2
**NF‐SD**	0.125	1
**Ciprofloxacin (CIP)**	1 μg mL^−1^	0.156 μg mL^−1^
**Cycloheximide (CHX)**	‐	0.0625 μg mL^−1^

Comparative analysis between fermented and non‐fermented solvent extracts revealed that the fermented CBS extract exhibited marginally better antimicrobial activity than non‐fermented CBS extracts against *S. mutans*. The solvent extraction of fermented CBS extracts, F‐A‐EtOH, F‐EtOH, F‐A‐MeOH, and F‐MeOH showed a lower MIC of 0.5 mg mL^−1^ against *S. mutans* than non‐fermented extracts, NF‐EtOAc, NF‐A‐EtOH, NF‐EtOH, NF‐A‐MeOH and NF‐MeOH, which had a MIC of 1 mg mL^−1^. Earlier literature suggested that non‐fermented cocoa extracts often exhibit superior antimicrobial activity due to higher diversity of phytochemicals compared with their fermented cocoa extracts. These studies are focused on comparing the antimicrobial activity of fermented and non‐fermented cocoa bean extract instead of CBS extracts. Smullen *et al*. reported that the non‐fermented cocoa bean extract with a MIC of 4 mg mL^−1^ demonstrated marginally better antibacterial activity against *S. mutans* than fermented cocoa, which had a MIC of 8 mg mL^−1^.^37^ Moreover, the non‐fermented cocoa bean extract was reported to show better antibacterial activity against *Porphyromonas gingivalis* than fermented cocoa.^38^ In contrast, the findings of the current study indicated that fermented CBS extracts exhibited greater inhibitory activity against *S. mutans* than non‐fermented CBS extracts. The enhanced activity might be attributed to the higher levels of TB and CAF present in the fermented extracts in comparison with the non‐fermented CBS extracts.

Previous studies on CBS have focused solely on using organic solvents as extractants. The aim of the current study was to investigate the antimicrobial potential of steam distillates of CBS, a method that has not been reported. In this study, steam distillation extracts, which contained volatile phytochemicals, exhibited antimicrobial activity three times greater than that of solvent‐extracted samples. The fermented steam distillation CBS extract also demonstrated superior antibacterial activity against *S. mutans* in comparison with its non‐fermented counterpart (MIC of F‐SD: 0.0625 μg mL^−1^; MIC of NF‐SD: 0.125 μg mL^−1^).

### Identification of Key Compounds for *S. mutans* Inhibition

**Figure 3:**
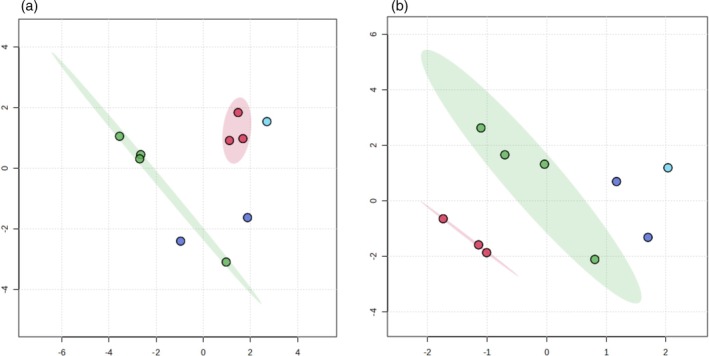
Data analysis using MetaboAnalyst software: (a) principal component analysis (PCA) and (b) partial least‐squares discriminant analysis (PLS‐DA) scores plot of metabolites from fermented (F‐EtOAc, F‐A‐EtOH, F‐EtOH, F‐A‐MeOH, and F‐MeOH) and non‐fermented extracts (NF‐EtOAc, NF‐A‐EtOH, NF‐EtOH, NF‐A‐MeOH, and NF‐MeOH). The plots distinguish between extracts based on their activity against *S. mutans*, categorized as active (minimum inhibitory concentration (MIC): 0.5 mg mL^−1^), moderately active (MIC: 1 mg mL^−1^), less active (MIC: 2 mg mL^−1^), and inactive (MIC: >2 mg mL^−1^).

It is essential to understand the specific phytochemicals responsible for the enhanced antimicrobial activity of fermented CBS extracts in order to evaluate their therapeutic potential. To identify the active compounds inhibiting *S. mutans*, the phytochemical compositions of CBS extracts were analyzed using LC‐QTOF‐MS and GC–MS. Inactive F‐EtOAc extracts were compared with active fermented and non‐fermented extracts using MetaboAnalyst. Unsupervised PCA and supervised PLS‐DA were performed (Fig. 3(a),(b), respectively) to classify non‐volatile bioactive compounds based on their metabolite profiles. Principal component analysis, which showed no clear group separation, explained 76% of the total variance across five components: PC1 (25.4%), PC2 (14.9%), PC3 (13.4%), PC4 (11.3%), and PC5 (10.7%). In contrast, PLS‐DA revealed separation of non‐volatile compounds among all extracts, with PC1 (11.5%), PC2 (17.2%), PC3 (16.7%), PC4 (8.8%), and PC5 (7.8%) accounting for a total variance of 62%. These findings indicate that the model effectively discriminated metabolites associated with varying levels of activity (active, moderately active, less active, and inactive) in fermented and non‐fermented solvent extracts. Principal component analysis and PLS‐DA analyses of volatile compounds from CBS steam distillation extracts (F‐SD and NF‐SD) were not performed due to the limited dataset, which comprised only two categories: highly active and less active.

**Figure 4:**
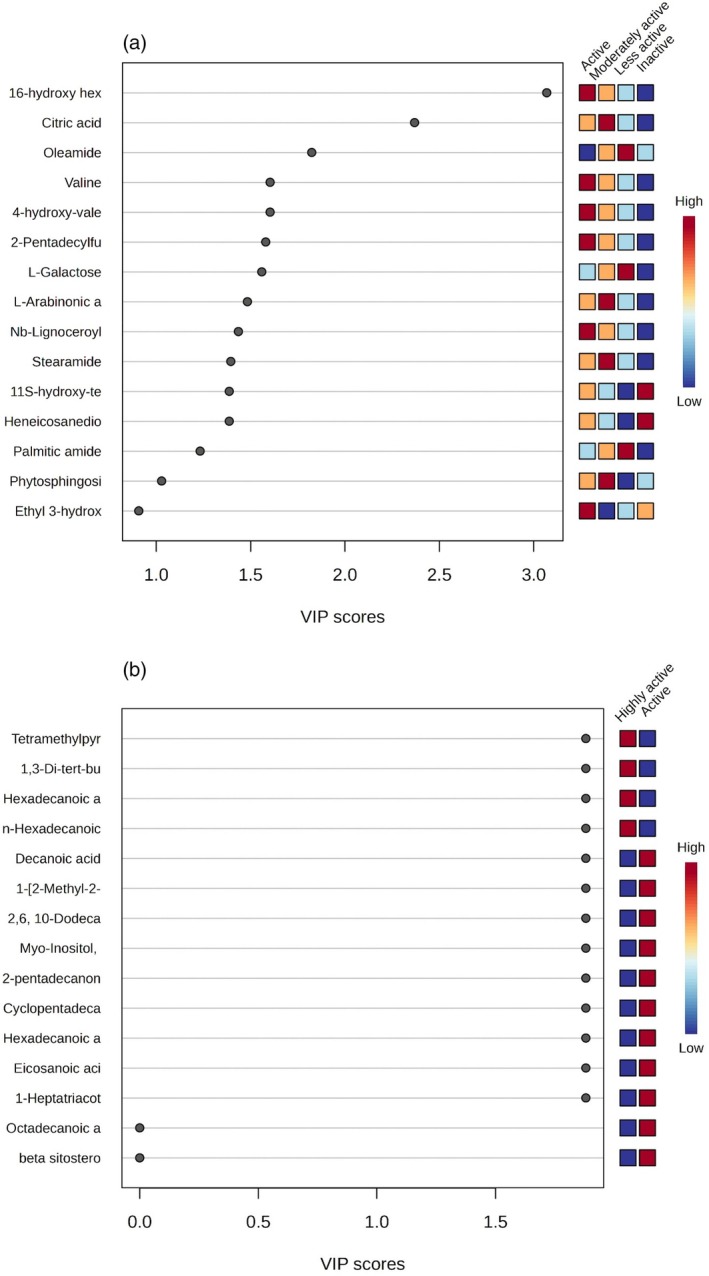
Data analysis using MetaboAnalyst software. The variable importance in projection (VIP) schematic scores of partial least‐squares discriminant analysis (PLS‐DA) analyses for (a) solvent cocoa bean shell (CBS) extracts (F‐EtOAc, F‐A‐EtOH, F‐EtOH, F‐A‐MeOH and F‐MeOH, NF‐EtOAc, NF‐A‐EtOH, NF‐EtOH, NF‐A‐MeOH and NF‐MeOH) and (b) steam distillation CBS extracts (F‐SD and NF‐SD). A high VIP score indicates greater discrimination between active and inactive samples against *S. mutans*. The color scale reflects metabolite abundance, with red representing higher abundance and blue representing lower abundance.

The variable importance in projection (VIP) scores, based on the PLS‐DA model of non‐volatile and volatile compounds, are reported in Fig. 4(a),(b). This estimates the importance of each metabolite in the PLS model. In this study, 16‐hydroxy hexadecanoic acid, observed only in the F‐EtOH, F‐A‐MeOH and F‐MeOH CBS extracts, was the most significant metabolite and might be associated with the inhibition of *S. mutans*. However, no prior studies have reported on 16‐hydroxy hexadecanoic acid (juniperic acid) possessing antimicrobial activity against *S. mutans* or any other pathogens. Despite that, structurally related compounds such as palmitic acid, stearic acid, lauric acid, caprylic acid, and 3‐hydroxyundecanoic acid have all demonstrated antimicrobial properties.^39^, ^40^, ^41^, ^42^ Further experimental validation is thus required to assess its significance accurately.

Based on the VIP score for volatile compounds, TMP, present only in F‐SD could potentially be one of the important metabolites that inhibit *S. mutans*. Tetramethylpyrazine has been reported for its anti‐inflammatory and anti‐cancer effects.^43^, ^44^, ^45^, ^46^ While numerous studies have reported on TMP's diverse biological activities, only limited research has investigated the analogs of TMP against *S. aureus*, *B. subtilis*, *S. agalactiae*, and *E. coli*.^47^, ^48^, ^49^ Moreover, the antibacterial activity of TMP against *S. mutans* has not been reported previously. In this study, TMP alone showed activity against *S. mutans* with MIC of 1.5 mg mL^−1^ which is 24‐fold higher than the MIC of steam distillation extract (F‐SD, MIC: 0.0625 mg mL^−1^), suggesting the synergistic effect of TMP with other compounds in the F‐SD extracts.

The weaker antibacterial activity of the NF‐SD extract against *S. mutans* might be due to the absence of TMP. NF‐SD contains more fatty acid esters and less free fatty acids, which are known to have stronger antibacterial properties, and this might also be one of the reasons for its weaker inhibition toward *S. mutans*. Studies indicated that the esterification of fatty acids tends to reduce antibacterial activity, except for esterified forms of sucrose.^50^ This study found that the NF‐SD extract mainly contains methylated and ethylated fatty acid esters with less free fatty acid than F‐SD.

The comparison of the key secondary metabolites (TB and CAF) in CBS waste showed that only TB significantly enhanced activity against *S. mutans*. Notably, TB exhibits substantial levels among the various extracts, with a false discovery rate (FDR) lower than 0.05, indicating its significance for antimicrobial activity. This is consistent with the results where TB (MIC: 1.25 mg mL^−1^) exhibited better antimicrobial activity against *S. mutans* than CAF (MIC: 4 mg mL^−1^), as shown in Table 2. Studies have also shown the antimicrobial effect of TB against caries‐associated bacteria, such as *S. mutans* and *Actinomyces naeslundii*, and non‐caries‐associated bacteria like *Lactobacillus acidophilus* and *E*. *faecalis*.^51^, ^52^

**Table 2:** The minimum inhibitory concentrations (MICs) of TB, CAF, and TMP against *S. mutans, C. albicans* and *A. baumannii* strains

Sample ID	Minimum inhibitory concentrations (mg mL^−1^)			
	*S. mutans* (ATCC 25175)	*C. albicans* (ATCC 10231)	*A. baumannii* (ATCC BAA 1605)	*A. baumannii* (C65)
**TB**	1.25	0.625	>1.25	1.25
**CAF**	4	4	2	2
**TMP**	1.5	>1.5	1.5	>1.5
**CIP**	1 μg mL^−1^	0.156 μg mL^−1^	>1 μg mL^−1^	0.256 μg mL^−1^
**CHX**.	‐	0.0625 μg mL^‐1^	‐	‐

Abbreviations: TB, theobromine; TMP, tetramethylpyrazine; CAF, caffeine; CIP, ciprofloxacin, CHX, cycloheximide.

Caffeine, TB and TMP showed activity against *A. baumannii*, with MICs of 2, 1.25 and 1.5 mg mL^−1^, respectively (Table 2). Although CAF has been reported to be active against *A. baumannii* at 4 mg mL^−1^, activity against *A. baumannii* by TB and TMP have not been reported previously.^53^ Thus, results from this study show the antimicrobial potential of CAF, TB and TMP. Their synergistic potential with CIP against various pathogens is discussed in the next section.

### Synergistic effects

The aim of the synergistic activity study was to assess the antimicrobial activity of common bioactive compounds in CBS like TB, CAF, and TMP against several microbes, alone or combined with CIP. Ciprofloxacin, a commonly prescribed antibiotic, was selected for the synergistic study due to its broad‐spectrum antimicrobial activity. However, resistance to CIP is a growing concern, which makes it an ideal candidate for synergy studies, as combining it with other bioactive compounds such as, CAF, TB and TMP could potentially help to restore its effectiveness, reduce the required dose, and potentially delay the development of resistance. The synergistic effect of TB, CAF, and TMP combined with CIP was determined by the FICI derived from the checkerboard synergism method. These synergistic effects were determined against a range of bacteria and fungi, including the ‘ESKAPE’ pathogens, *B. cereus, E. faecalis, B. subtilis, S. mutans, E. coli*, and *C. albicans* as outlined in Table 3.

**Table 3:** The combined effects of TB, TMP and CAF with CIP against various microbial strains, as determined by the checkerboard method

Bacteria	Minimum inhibitory concentrations (MIC)				Fractional inhibitory concentration index (FICI)	Combination effect
	CBS bioactive mg mL^−1^		CIP μg mL^−1^	Mixed μg mL^−1^		
MRSA	TB	>1.25	0.5	0.5	1.00	Indifference
ATCC 33591	CAF	16		>0.5	1.00	Indifference
	TMP	>1.5		0.5	1.00	Indifference
MSSA	TB	>1.25	0.004	0.004	1.00	Indifference
ATCC 25923	CAF	16		0.004	1.00	Indifference
	TMP	>1.5		0.004	1.00	Indifference
MSSA	TB	>1.25	0.002	0.002	1.00	Indifference
6538	CAF	16		0.002	1.00	Indifference
	TMP	>1.5		0.002	1.00	Indifference
*K. pneumoniae*	TB	>1.25	0.256	0.256	1.00	Indifference
ATCC 10031	CAF	8		0.256	1.00	Indifference
	TMP	>1.5		0.512	2.00	Indifference
*E. faecalis*	TB	>1.25	1	1	1.00	Indifference
ATCC 29212	CAF	8		1	1.00	Indifference
	TMP	>1.5		1	1.00	Indifference
*E. faecium*	TB	>1.25	0.032	0.032	1.00	Indifference
ATCC 700221	CAF	4		0.032	1.00	Indifference
	TMP	1.5		0.032	1.00	Indifference
*C. albicans*	TB	0.625	CHX	0.625	1.00	Indifference
10 231	CAF	4	0.625	0.625	1.00	Indifference
	TMP	>1.5		0.313	0.50	Synergy
*B. cereus*	TB	>1.25	0.128	0.256	2.00	Indifference
ATCC 14579	CAF	8		0.256	2.00	Indifference
	TMP	>1.5		0.256	2.00	Indifference
*P. aeruginosa*	TB	>1.25	0.256	0.256	1.00	Indifference
ATCC 10145	CAF	>16		0.128	0.50	Synergy
	TMP	>1.5		0.256	1.00	Indifference
*A. baumannii*	TB	>1.25	>0.512	>0.512	1.00	Indifference
BAA 1605	CAF	2		>0.512	1.00	Indifference
	TMP	1.5		>0.512	1.00	Indifference
*A. baumannii*	TB	1.25	0.256	>0.512	2.00	Indifference
C65	CAF	2		0.256	1.00	Indifference
	TMP	>1.5		0.256	1.00	Indifference
*A. baumannii*	TB	>1.25	0.128	0.128	1.00	Indifference
C28	CAF	2		0.128	1.00	Indifference
	TMP	>1.5		0.128	1.00	Indifference
*A. baumannii*	TB	>1.25	0.256	0.256	1.00	Indifference
C98	CAF	4		0.256	1.00	Indifference
	TMP	>1.5		0.256	1.00	Indifference
*E. coli*	TB	>1.25	0.016	0.016	1.00	Indifference
ATCC 10798	CAF	4		0.016	1.00	Indifference
	TMP	>1.5		0.016	1.00	Indifference
*S. mutans*	TB	1.25	1	1	1.00	Indifference
ATCC 25175	CAF	4		1	1.00	Indifference
	TMP	1.5		1	1.00	Indifference
*B. subtilis*	TB	1.25	0.256	0.125	0.48	Synergy
ATCC 8188	CAF	16		0.256	1.00	Indifference
	TMP	>1.5		0.256	1.00	Indifference
*K. aerogenes*	TB	1.25	0.5	0.0625	0.31	Synergy
ATCC 13048	CAF	16		0.0625	0.25	Synergy
	TMP	>1.5		0.125	0.38	Synergy

Abbreviations: TB, theobromine; TMP, tetramethylpyrazine; CAF, caffeine; CIP, ciprofloxacin; CBS, cocoa bean shell; MSSA, Methicillin‐susceptible S. aureus; MRSA, Methicillin‐resistant S. aurues.

Multiple synergistic relationships were noted between CIP/CHX and common compounds found in CBS waste. It is interesting to note that a synergistic effect was observed when CIP was combined with all three tested metabolites found in CBS (TB, CAF, and TMP) against *K. aerogenes*. This finding is significant as *K. aerogenes* is one of the most virulent pathogens and has displayed resistance to multiple antibiotics.

Similarly, synergy was observed between TMP and CHX against *C. albicans*, and between CAF and CIP against *P. aeruginosa*. Theobromine and CIP also demonstrated a positive synergistic effect against *B. subtilis*. The mechanisms underlying these interactions are not yet understood and this warrants further investigation.

## CONCLUSION

Both fermented and non‐fermented CBS extracts were found to exhibit antimicrobial activity against *S. mutans* and *C. albicans* with fermented extracts showing better activity. Steam distilled extracts also showed better antibacterial activity than solvent extracts. Using a metabolomic approach, TMP was identified as a potential antibacterial agent against *S. mutans*. The results indicated that the composition of fatty acid esters and free fatty acids impacts the antimicrobial efficacy significantly. The stronger antibacterial activity of fermented steam distillation extracts could be attributed to the higher content of free fatty acids, with less methylated and ethylated fatty acid. A positive synergistic effect was also observed when CAF, TB, and TMP were combined with CIP against *K. aerogenes* but remained indifferent against most of the other bacterial and fungal strains tested. Synergy was also observed when TMP was combined with CHX against *C. albicans*. Caffeine and CIP also worked synergistically against *P. aeruginosa* while TB and CIP also showed positive synergy against *B. subtilis*.

In conclusion, this study shows the complex interplay of various metabolites found in fermented and non‐fermented CBS extracts and their contributions to antimicrobial activity. Although some of the key bioactive compounds have been identified, the lack of structural confirmation by NMR spectroscopy or further analytical data limits a more conclusive analysis of the proposed compounds. The mechanism of action also remains unclear. Future studies could thus focus on targeted metabolic or enzymatic analyses to elucidate the mode of action of these bioactive molecules.

## AUTHOR CONTRIBUTIONS

Yie Kie Chong: investigation; formal analysis; writing – original draft. Wei Khang Gan: investigation; formal analysis; writing – original draft. Joash Ban Lee Tan: writing – review and editing. Ahmad Kamil Hj Mohd Jaaffar: writing – review and editing. Zainal Baharum: writing – review and editing. Keng Yoon Yeong: conceptualization, supervision, writing – review and editing. All authors have read and agreed to the published version of the manuscript.

## CONFLICT OF INTEREST

The authors declare no conflict of interest.

## Supporting information


**Data S1.** Supporting Information.

## Data Availability

The data that support the findings of this study are available from the corresponding author upon reasonable request.

## References

[1] Endale H , Mathewos M and Abdeta D , Potential causes of spread of antimicrobial resistance and preventive measures in one health perspective—a review. Infect Drug Resist 16:7515–7545 (2023). 38089962 10.2147/IDR.S428837 PMC10715026

[2] Mármol I , Quero J , Ibarz R , Ferreira‐Santos P , Teixeira JA , Rocha CM *et al*., Valorization of agro‐food by‐products and their potential therapeutic applications. Food Bioprod Process 128:247–258 (2021).

[3] Tan BL and Norhaizan ME , Rice by‐products: phytochemicals and food products application. Springer International Publishing, Cham (2020).

[4] Nani M and Krishnaswamy K , A natural whitening alternative from upcycled food waste (acid whey) and underutilized grains (millet). Sci Rep 13:6482 (2023). 37081016 10.1038/s41598-023-32204-4 PMC10119097

[5] dos Santos ÉM , de Macedo LM , Tundisi LL , Ataide JA , Camargo GA , Alves RC *et al*., Coffee by‐products in topical formulations: a review. Trends Food Sci Technol 111:280–291 (2021).

[6] Choudhary P , Devi TB , Tushir S , Kasana RC and Popatrao DS , Mango seed kernel: a bountiful source of nutritional and bioactive compounds. Food Bioprocess Technol 16:289–312 (2023).

[7] Belwal T , Cravotto C , Ramola S , Thakur M , Chemat F and Cravotto G , Bioactive compounds from cocoa husk: extraction, analysis and applications in food production chain. Foods 11:798 (2022). 35327221 10.3390/foods11060798 PMC8947495

[8] Ministry of Plantation and Commodities (KPK) , National biomass action plan 2023–2030.

[9] Rojo‐Poveda O , Barbosa‐Pereira L , Zeppa G and Stévigny C , Cocoa bean shell—a by‐product with nutritional properties and biofunctional potential. Nutrients 12:1123 (2020). 32316449 10.3390/nu12041123 PMC7230451

[10] Rojo‐Poveda O , Barbosa‐Pereira L , Orden D , Stévigny C , Zeppa G and Bertolino M , Physical properties and consumer evaluation of cocoa bean shell‐functionalized biscuits adapted for diabetic consumers by the replacement of sucrose with tagatose. Foods 9:814 (2020). 32575809 10.3390/foods9060814 PMC7353579

[11] Mellinas AC , Jiménez A and Garrigós MC , Optimization of microwave‐assisted extraction of cocoa bean shell waste and evaluation of its antioxidant, physicochemical and functional properties. LWT 127:109361 (2020).

[12] Rojo‐Poveda O , Ribeiro SO , Anton‐Sales C , Keymeulen F , Barbosa‐Pereira L , Delporte C *et al*., Evaluation of cocoa bean shell antimicrobial activity: a tentative assay using a metabolomic approach for active compound identification. Planta Med 87:841–849 (2021). 10.1055/a-1499-7829. 34020491

[13] Matsumoto M , Tsuji M , Okuda J , Sasaki H , Nakano K , Osawa K *et al*., Inhibitory effects of cacao bean husk extract on plaque formation in vitro and in vivo. Eur J Oral Sci 112:249–252 (2004). 15154923 10.1111/j.1600-0722.2004.00134.x

[14] Ooshima T , Osaka Y , Sasaki H , Osawa K , Yasuda H , Matsumura M *et al*., Caries inhibitory activity of cacao bean husk extract in in‐vitro and animal experiments. Arch Oral Biol 45:639–645 (2000). 10869475 10.1016/s0003-9969(00)00042-x

[15] Sakagami H and Matsuta T , Biological activity of cacao husk and mass lignin‐carbohydrate complexes, in Chocolate in health and nutrition. Humana Press, Totowa, NJ, pp. 247–262 (2013).

[16] Rossin D , Barbosa‐Pereira L , Iaia N , Testa G , Sottero B , Poli G *et al*., A dietary mixture of oxysterols induces in vitro intestinal inflammation through TLR2/4 activation: the protective effect of cocoa bean shells. Antioxidants 8:151 (2019). 31151323 10.3390/antiox8060151 PMC6617147

[17] Ruesgas‐Ramón M , Figueroa‐Espinoza MC , Durand E , Suárez‐Quiroz ML , González‐Ríos O , Rocher A *et al*., Identification and quantification of phytoprostanes and phytofurans of coffee and cocoa by‐ and co‐products. Food Funct 10:6882–6891 (2019). 31584595 10.1039/c9fo01528k

[18] Rebollo‐Hernanz M , Zhang Q , Aguilera Y , Martín‐Cabrejas MA and de Mejia EG , Cocoa shell aqueous phenolic extract preserves mitochondrial function and insulin sensitivity by attenuating inflammation between macrophages and adipocytes in vitro. Mol Nutr Food Res 63:1801413 (2019). 10.1002/mnfr.201801413 31018035

[19] Aprotosoaie AC , Luca SV and Miron A , Flavor chemistry of cocoa and cocoa products—an overview. Compr Rev Food Sci Food Saf 15:73–91 (2016). 33371573 10.1111/1541-4337.12180

[20] Voigt J , Textoris‐Taube K and Wöstemeyer J , pH‐dependency of the proteolytic formation of cocoa‐ and nutty‐specific aroma precursors. Food Chem 255:209–215 (2018). 29571468 10.1016/j.foodchem.2018.02.045

[21] Barišić V , Kopjar M , Jozinović A , Flanjak I , Ačkar Đ , Miličević B *et al*., The chemistry behind chocolate production. Molecules 24:3163 (2019). 31480281 10.3390/molecules24173163 PMC6749277

[22] Baharum Z , Rahimudin AN , Suzielawanis Ismail R , Abdul Karim A and Azilah Abdullah N , Formulation and evaluation of cocoa antibacterial night cream from cocoa shell extract of Theobroma cacao for skincare. Malaysian Cocoa J 11:121–128 (2019).

[23] Apriyanto M , Study on effect of fermentation to the quality parameter of cocoa bean in Indonesia. Asian J Dairy Food Res 35:160–163 (2016).

[24] Hernández‐Hernández C , Viera‐Alcaide I , Morales‐Sillero AM , Fernández‐Bolaños J and Rodríguez‐Gutiérrez G , Bioactive compounds in Mexican genotypes of cocoa cotyledon and husk. Food Chem 240:831–839 (2018). 28946348 10.1016/j.foodchem.2017.08.018

[25] Mudenuti NV d R , de Camargo AC , de Alencar SM , Danielski R , Shahidi F , Madeira TB *et al*., Phenolics and alkaloids of raw cocoa nibs and husk: the role of soluble and insoluble‐bound antioxidants. Food Biosci 42:101085 (2021).

[26] Pang Z , Lu Y , Zhou G , Hui F , Xu L , Viau C *et al*., MetaboAnalyst 6.0: towards a unified platform for metabolomics data processing, analysis and interpretation. Nucleic Acids Res 52:398–402 (2024). 10.1093/nar/gkae253 PMC11223798 38587201

[27] Wayne PA , CLSI Supplement M100, 30th ed. Clinical and Laboratory Standards Institute. CLSI. Standards for antimicrobial susceptibility testing (2020).

[28] Muzahid NH , Hussain MH , Huët MA , Dwiyanto J , Su TT , Reidpath D *et al*., Molecular characterization and comparative genomic analysis of Acinetobacter baumannii isolated from the community and the hospital: An epidemiological study in Segamat. MalaysiaMicrob Genome 9:977 (2023). 10.1099/mgen.0.000977 PMC10210948 37018035

[29] Bellio P , Fagnani L , Nazzicone L and Celenza G , New and simplified method for drug combination studies by checkerboard assay. MethodsX 8:101543 (2021). 34754811 10.1016/j.mex.2021.101543 PMC8563647

[30] Ahmad A , Van Vuuren S and Viljoen A , Unravelling the complex antimicrobial interactions of essential oils‐The case of Thymus vulgaris (thyme). Molecules 19:2896–2910 (2014). 24662066 10.3390/molecules19032896 PMC6271043

[31] Krähmer A , Engel A , Kadow D , Ali N , Umaharan P , Kroh LW *et al*., Fast and neat – determination of biochemical quality parameters in cocoa using near infrared spectroscopy. Food Chem 181:152–159 (2015). 25794734 10.1016/j.foodchem.2015.02.084

[32] Calvo AM , Botina BL , García MC , Cardona WA , Montenegro AC and Criollo J , Dynamics of cocoa fermentation and its effect on quality. Sci Rep 11:16746 (2021). 34408194 10.1038/s41598-021-95703-2 PMC8373873

[33] Zhang Y , Wang Y , Zhang F , Wang K , Liu G , Yang M *et al*., Allyl methyl disulfide inhibits IL‐8 and IP‐10 secretion in intestinal epithelial cells via the NF‐κB signaling pathway. Int Immunopharmacol 27:156–163 (2015). 26003845 10.1016/j.intimp.2015.05.013

[34] Aprotosoaie A , Miron A , Trifan A , Luca V and Costache II , The cardiovascular effects of cocoa polyphenols—An overview. Diseases 4:39 (2016). 28933419 10.3390/diseases4040039 PMC5456324

[35] Todorovic V , Milenkovic M , Vidovic B , Todorovic Z and Sobajic S , Correlation between antimicrobial, antioxidant activity, and polyphenols of alkalized/nonalkalized cocoa powders. J Food Sci 82:1020–1027 (2017). 28272800 10.1111/1750-3841.13672

[36] Santos RX , Oliveira DA , Sodré GA , Gosmann G , Brendel M and Pungartnik C , Antimicrobial activity of fermented Theobroma cacao pod husk extract. Genet Mol Res 13:7725–7735 (2014). 25299086 10.4238/2014.September.26.10

[37] Smullen J , Koutsou GA , Foster HA , Zumbé A and Storey DM , The antibacterial activity of plant extracts containing polyphenols against Streptococcus mutans. Caries Res 41:342–349 (2007). 17713333 10.1159/000104791

[38] Maajid MN , Sutjiati R and Joelijanto R , Antibacterial activity of fermented and non‐fermented edel cacao bean extract (*Theobroma cacao* L.) against Porphyromonas gingivalis. Health Notions 5:289–294 (2021).

[39] Thammasut W , Intaraphairot T , Chantadee T , Senarat S , Patomchaiviwat V , Chuenbarn T *et al*., Antimicrobial and antitumoral activities of saturated fatty acid solutions. Mater Today Proc (2023).

[40] Joujou FM , Darra NE , Rajha HN , Sokhn ES and Alwan N , Evaluation of synergistic/antagonistic antibacterial activities of fatty oils from apricot, date, grape, and black seeds. Sci Rep 14:6532 (2024). 38503788 10.1038/s41598-024-54850-y PMC10951330

[41] Casillas‐Vargas G , Ocasio‐Malavé C , Medina S , Morales‐Guzmán C , Del Valle RG , Carballeira NM *et al*., Antibacterial fatty acids: an update of possible mechanisms of action and implications in the development of the next generation of antibacterial agents. Prog Lipid Res 82:101093 (2021). 33577909 10.1016/j.plipres.2021.101093 PMC8137538

[42] Guimarães A and Venâncio A , The potential of fatty acids and their derivatives as antifungal agents: a review. Toxins (Basel) 14:188 (2022). 35324685 10.3390/toxins14030188 PMC8954725

[43] Liu N , Lin L , Wang JQ , Zhang FK and Wang JP , Tetramethylpyrazine supplementation reduced Salmonella Typhimurium load and inflammatory response in broilers. Poult Sci 98:3158–3164 (2019). 30895324 10.3382/ps/pez128

[44] Han X , Chen X , Chen S , Luo Q , Liu X , He A *et al*., Tetramethylpyrazine attenuates endotoxin‐induced retinal inflammation by inhibiting microglial activation via the TLR4/NF‐κB signalling pathway. Biomed Pharmacother 128:110273 (2020). 32460188 10.1016/j.biopha.2020.110273

[45] Hou YX , Ren W , He QQ , Huang LY , Gao TH and Li H , Tetramethylpyrazine induces reactive oxygen species‐based mitochondria‐mediated apoptosis in colon cancer cells. World J Gastroint Oncol 17:104922 (2025). 10.4251/wjgo.v17.i4.104922. PMC11995317 40235896

[46] Yang SJ , Wu SD , Dai WL , Pang LW , Xie YF , Ren TQ *et al*., Tetramethylpyrazine: a review of its antitumor potential and mechanisms. Front Pharmacol 12:764331 (2021). 34975475 10.3389/fphar.2021.764331 PMC8716857

[47] Bassin J , Botha M , Garikipati R , Goyal M , Martin L and Shah AS , Synthesis and antibacterial activity of benzo[4,5]isothiazolo[2,3‐a]pyrazine‐6,6‐dioxide derivatives. Molecules 22:1889 (2017). 29113049 10.3390/molecules22111889 PMC6150388

[48] Foks H , Pancechowska‐Ksepko D , Kędzia A , Zwolska Z , Janowiec M and Augustynowicz‐Kopeć E , Synthesis and antibacterial activity of 1H‐pyrazolo[3,4‐b]pyrazine and ‐pyridine derivatives. II Farmaco 60:513–517 (2005). 10.1016/j.farmac.2005.05.002 15950227

[49] Bonde CG and Gaikwad NJ , Synthesis and preliminary evaluation of some pyrazine containing thiazolines and thiazolidinones as antimicrobial agents. Bioorg Med Chem 12:2151–2161 (2004). 15080915 10.1016/j.bmc.2004.02.024

[50] McGaw LJ , Jäger AK and van Staden J , Antibacterial effects of fatty acids and related compounds from plants. S Afr J Bot 68:417–423 (2002).

[51] Rafiq IH , Dame‐Teixeira N and Do T , The antimicrobial activity of theobromine against cariogenic microbes: an in vitro pilot study. BDJ open 10:8 (2024). 38302447 10.1038/s41405-024-00190-y PMC10834536

[52] Lakshmi A , Vishnurekha C and Baghkomeh PN , Effect of theobromine in antimicrobial activity: an: in vitro: study. Dent Res J 16:76–80 (2019). PMC6364351 30820200

[53] Woziwodzka A , Krychowiak‐Maśnicka M , Gołuński G , Łosiewska A , Borowik A , Wyrzykowski D *et al*., New life of an old drug: caffeine as a modulator of antibacterial activity of commonly used antibiotics. Pharmaceuticals 15:872 (2022). 35890171 10.3390/ph15070872 PMC9315996

