# Supplementary material for: Exploring the antimicrobial activity of fermented and non‐fermented cocoa bean shell extracts through metabolomics analysis and synergistic studies
Source: J Sci Food Agric. 2025 May 13;105(12):6495–505. doi: 10.1002/jsfa.14366 (PMC12355342; doi:10.1002/jsfa.14366)
Supplement: Supplementary file 1 — Data S1. Supporting Information. [file JSFA-105-6495-s001.docx]

**Supporting Information**

**Table S1:** The LC-QTOF-MS volume intensity (area abundance) of compounds in fermented and non-fermented CBS extracts obtained via solvent extractions.

| **Compounds** | **Volume Intensity** | | | | | | | | | |
| --- | --- | --- | --- | --- | --- | --- | --- | --- | --- | --- |
|  | **F-EtOAc** | **F-A-EtOH** | **F-EtOH** | **F-A-MeOH** | **F-MeOH** | **NF-EtOAc** | **NF-A-EtOH** | **NF-EtOH** | **NF-A-MeOH** | **NF-MeOH** |
| 10,11-dihydroxy stearic acid | 113882 | 0 | 0 | 0 | 0 | 0 | 0 | 0 | 0 | 685656 |
| 11S-hydroxy-tetradecanoic acid | 118065 | 0 | 0 | 0 | 0 | 0 | 0 | 0 | 0 | 0 |
| 14-hydroxy stearic acid | 0 | 225641 | 0 | 148740 | 0 | 0 | 0 | 0 | 0 | 0 |
| 16-hydroxy hexadecanoic acid | 0 | 0 | 4164707 | 2685844 | 3137405 | 0 | 0 | 0 | 0 | 0 |
| 16Z-octadecenoic acid | 0 | 0 | 0 | 0 | 0 | 0 | 0 | 0 | 0 | 1049571 |
| L-valinol | 0 | 2914679 | 3029105 | 0 | 0 | 0 | 0 | 0 | 3082154 | 1086669 |
| 2-Hydroxyhexadecanoic acid | 0 | 0 | 0 | 0 | 0 | 0 | 4024810 | 7162176 | 4359084 | 0 |
| 2-Methyl-2-(methyldithio)propanal | 0 | 0 | 0 | 0 | 0 | 0 | 332846 | 0 | 0 | 0 |
| 2-Pentadecylfuran | 0 | 0 | 733852 | 0 | 0 | 0 | 0 | 0 | 0 | 0 |
| 3,4-Dihydroxybenzoic acid | 170927 | 0 | 0 | 0 | 273006 | 0 | 0 | 0 | 0 | 0 |
| 3,9-Dimethyluric acid | 0 | 0 | 0 | 0 | 0 | 0 | 0 | 0 | 0 | 4848618 |
| 3-Aminoquinoline | 0 | 0 | 0 | 0 | 0 | 2357242 | 0 | 0 | 0 | 0 |
| 4-Caffeoyl-1,5-quinolactone | 0 | 0 | 0 | 0 | 0 | 0 | 327917 | 186654 | 256291 | 377781 |
| 4-hydroxy-valeric acid | 0 | 0 | 0 | 0 | 228525 | 0 | 0 | 0 | 0 | 0 |
| 5,3'-Dihydroxy-4',5'-dimethoxy-6,7-methylenedioxyisoflavone | 0 | 0 | 0 | 0 | 0 | 0 | 0 | 108621 | 0 | 0 |
| Linolenic acid | 0 | 0 | 0 | 0 | 0 | 0 | 0 | 0 | 0 | 1490183 |
| C16 Sphinganine | 20011850 | 0 | 23801528 | 0 | 23967214 | 0 | 26419982 | 26089550 | 25378774 | 0 |
| Caffeine | 4123804 | 2217496 | 4234317 | 0 | 3424038 | 2453122 | 2081670 | 765580 | 1478922 | 1895104 |
| Caffeoyl aspartic acid | 0 | 309088 | 0 | 0 | 307446 | 0 | 969066 | 0 | 866935 | 753677 |
| Citric acid | 0 | 422224 | 169759 | 512209 | 412289 | 0 | 2979554 | 0 | 5739226 | 983527 |
| D-1-[(3-Carboxypropyl)amino]-1-deoxyfructose | 0 | 0 | 0 | 0 | 0 | 0 | 423239 | 0 | 0 | 0 |
| Dehydroascorbic acid | 0 | 0 | 0 | 0 | 0 | 0 | 241986 | 0 | 0 | 0 |
| D-Glucuronic acid | 0 | 94768 | 0 | 0 | 0 | 0 | 0 | 0 | 506043 | 330786 |
| D-Sorbitol | 2064534 | 1255655 | 5258480 | 705206 | 0 | 0 | 0 | 0 | 0 | 323782 |
| D-α-Hydroxyglutaric acid | 0 | 80768 | 0 | 0 | 75256 | 0 | 0 | 49271 | 220560 | 250183 |
| Epifisetinidol-4alpha-ol | 0 | 257464 | 148446 | 0 | 0 | 716690 | 4248990 | 1921758 | 3104942 | 2497797 |
| Ethyl 3-hydroxybutyrate | 460826 | 315612 | 538067 | 233936 | 694643 | 0 | 0 | 0 | 0 | 0 |
| Glucoheptonic acid | 0 | 110928 | 135386 | 88163 | 0 | 35123 | 0 | 587134 | 0 | 0 |
| Glutarylglycine | 0 | 268907 | 0 | 274658 | 0 | 0 | 0 | 0 | 0 | 0 |
| Glyceric acid | 0 | 66358 | 0 | 0 | 0 | 0 | 0 | 0 | 0 | 0 |
| Heneicosanedioic acid | 1602960 | 0 | 0 | 0 | 0 | 0 | 0 | 0 | 0 | 0 |
| Kaempferol 3-(2''-(Z)-p-coumaroylglucoside) | 0 | 0 | 0 | 0 | 0 | 91379 | 0 | 0 | 0 | 0 |
| L-Arabinonic acid | 0 | 0 | 178111 | 0 | 0 | 0 | 0 | 0 | 171808 | 150553 |
| Levoglucosan | 72042 | 0 | 0 | 338404 | 0 | 0 | 0 | 0 | 0 | 0 |
| L-Galactono-1,4-lactone | 0 | 0 | 0 | 0 | 0 | 0 | 0 | 183323 | 0 | 0 |
| L-Galactose | 0 | 265972 | 450817 | 209580 | 564021 | 538424 | 989318 | 1458616 | 0 | 2028716 |
| N-[4'-hydroxy-(E)-cinnamoyl]-L-aspartic acid | 0 | 0 | 0 | 0 | 0 | 0 | 223343 | 0 | 0 | 0 |
| Nb-Lignoceroyltryptamine | 0 | 0 | 0 | 6864302 | 0 | 0 | 0 | 0 | 0 | 0 |
| Oleamide | 3391086 | 41636948 | 0 | 0 | 6091185 | 23080314 | 0 | 6495671 | 3934551 | 3685190 |
| Oleoyl Ethyl Amide | 0 | 0 | 0 | 0 | 0 | 1598032 | 0 | 0 | 0 | 0 |
| Palmitic amide | 0 | 5529607 | 0 | 0 | 0 | 4644876 | 0 | 1115054 | 0 | 0 |
| Phytosphingosine | 2886200 | 0 | 3089279 | 1875647 | 3066484 | 4329004 | 3358490 | 4258326 | 3290547 | 4769514 |
| Proanthocyanidin A1 | 1006517 | 0 | 0 | 2115161 | 1047108 | 0 | 1027683 | 1810010 | 1126011 | 1335355 |
| Robinetinidol-(4alpha->8)-catechin-(6->4alpha)-robinetinidol | 0 | 0 | 0 | 0 | 0 | 0 | 710930 | 294122 | 627915 | 601795 |
| Stearamide | 0 | 0 | 0 | 0 | 1460103 | 5655212 | 0 | 0 | 0 | 1031728 |
| Theobromine | 32080726 | 16888034 | 11367764 | 18206058 | 15249392 | 7890222 | 9281147 | 2955620 | 6690208 | 12900384 |
| Valine | 0 | 0 | 0 | 0 | 1293974 | 0 | 0 | 0 | 0 | 0 |
| Xanthine | 0 | 74592 | 0 | 0 | 0 | 0 | 0 | 0 | 0 | 0 |
| Xylitol | 0 | 0 | 0 | 0 | 0 | 0 | 0 | 73077 | 0 | 0 |
| α-9(10)-EpODE | 0 | 0 | 0 | 0 | 0 | 0 | 0 | 0 | 0 | 1036604 |


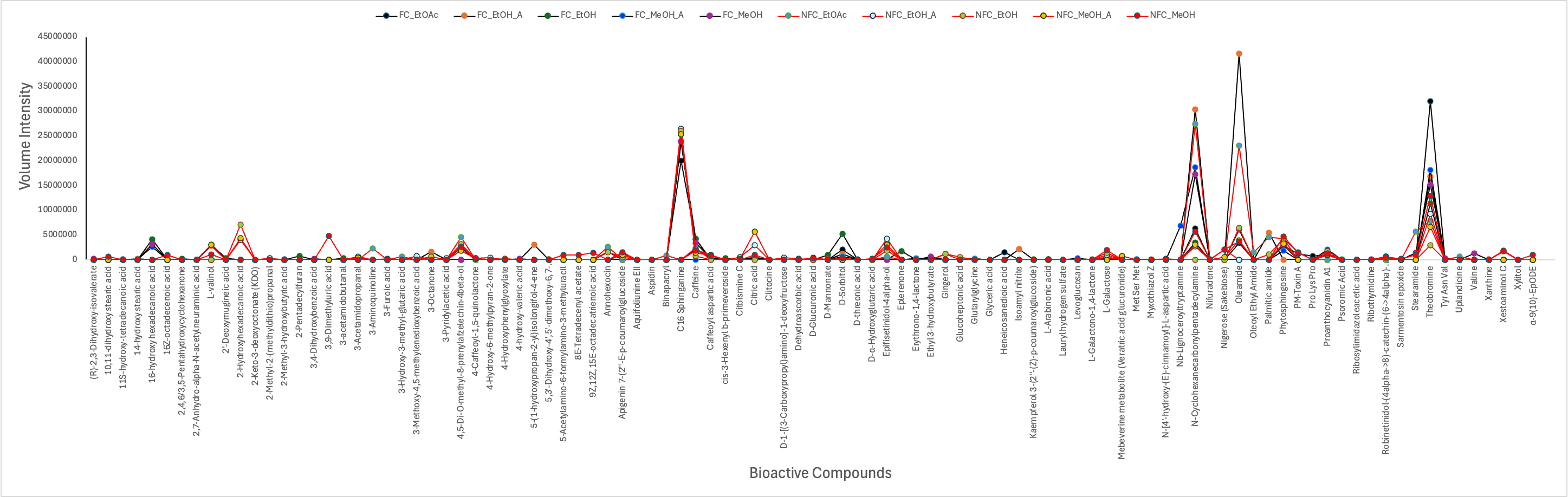


**Figure S1:** The line graph of volume intensity determined by LCMS of each bioactive compounds in the fermented and non-fermented CBS extracts.

**Table S2:** The GCMS volume intensity (area abundance) of volatile compounds in fermented (F-SD) and non-fermented (NF-SD) CBS extracts obtained via steam distillation.

| **Volatile compounds** | **Volume Intensity** | |
| --- | --- | --- |
|  | **F-SD** | **NF-SD** |
| Tetramethylpyrazine | 1764060 | 0 |
| Dodecane | 1071827 | 0 |
| 1,3-Di-tert-butylbenzene | 1597813 | 0 |
| Tetradecane | 267952 | 0 |
| Hexadecane | 1434146 | 0 |
| Eicosane | 5219672 | 0 |
| n-Dodecanoic acid | 1098663 | 125933 |
| Heptadecane | 470082 | 0 |
| Heneicosane | 1871756 | 0 |
| Methyl palmitate | 769745 | 0 |
| Hexadecanoic acid | 301157 | 0 |
| Ethyl palmitate | 859499 | 0 |
| Methyl oleate | 1524190 | 0 |
| Ethyl oleate | 2303371 | 972694 |
| n-Hexadecanoic acid | 4241982 | 1672718 |
| 9-Octadecenoic acid | 1965006 | 0 |
| Phenylethyl alcohol | 0 | 582956 |
| Octanoic acid | 0 | 390091 |
| Undecane | 0 | 58666 |
| Nonanoic acid | 0 | 89501 |
| Decanoic acid | 0 | 181003 |
| Isopentyl benzoate | 0 | 100591 |
| 1-undecene | 0 | 77886 |
| Z-1,6-Tridecadiene | 0 | 95321 |
| 1-[2-Methyl-2-(4-methyl-3-pentenyl)cyclopropyl]ethanol | 0 | 26378 |
| Decanoic acid ethyl ester | 0 | 123027 |
| N-(Trifluoroacetyl)-N,O,O',O''-tetrakis(trimethylsilyl)norepinephrine | 0 | 28764 |
| 1-pentadecene | 0 | 43799 |
| 2-Nonadecanone | 0 | 412447 |
| 3-methyldiadamantane | 0 | 17989 |
| Decanoic acid methyl ester | 0 | 1619 |
| 2,6, 10-Dodecatrien-1-ol,3,7,11,trimethyl-, (Z,E)- | 0 | 27410 |
| Myo-Inositol, 4-C-methyl | 0 | 21525 |
| 2-pentadecanone, 6,10,14-trimethyl- | 0 | 204464 |
| n-Octylidencyclohexane | 0 | 222363 |
| Hexadecanoic acid methyl ester | 0 | 1610438 |
| Cyclopentadecanone, 2-hydroxyl- | 0 | 135553 |
| Ethyl 9-hexadecenoate | 0 | 2016040 |
| Hexadecanoic acid ethyl ester | 0 | 1435665 |
| Heneicosanoic acid methyl ester | 0 | 36668 |
| 3-cyclohexene-1-methanol, alpha., 4-dimethyl-.alpha.-(4-dimethyl-3-pentenyl)-[R-(R*,R*)]- | 0 | 53383 |
| 9-Octadecenoic acid, methyl ester, (E)- | 0 | 401077 |
| Octadecanoic acid, methyl ester | 0 | 377703 |
| Eicosanoic acid ethyl ester | 0 | 51053 |
| beta sitosterol | 0 | 248426 |
| 1-Heptatriacotanol | 0 | 29358 |


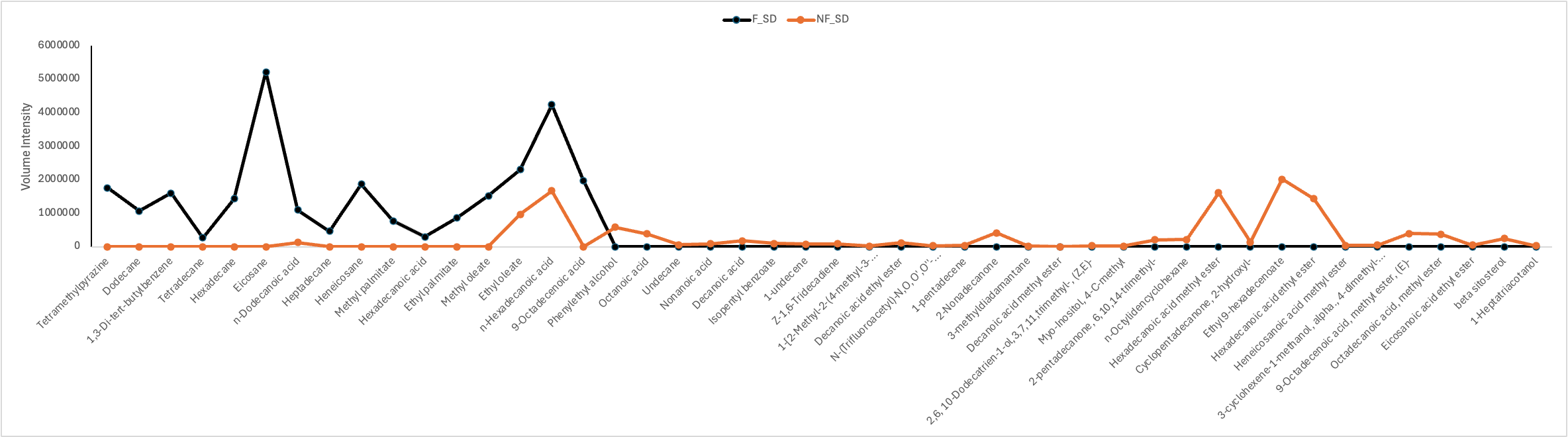
**Figure S2:** The line graph of volume intensity determined by GCMS of each bioactive compounds in the fermented and non-fermented CBS steam distillation extracts.

**Table S3:** Minimum inhibitory concentration (MIC) of CBS extracts against selected bacterial and fungal strains.

| **Bacteria** | **Minimum inhibitory concentration, MIC (mg/mL)** | | | | | | | | | | | | |
| --- | --- | --- | --- | --- | --- | --- | --- | --- | --- | --- | --- | --- | --- |
|  | **F-EtOAc** | **F-A-EtOH** | **F-EtOH** | **F-A-MeOH** | **F-MeOH** | **NF-EtOAc** | **NF-A-EtOH** | **NF-EtOH** | **NF-A-MeOH** | **NF-MeOH** | **F-SD** | **NF-SD** | **CIP*** |
| MSSA | >1 | >5 | >5 | >2.5 | >5 | >5 | >5 | >5 | >5 | >5 | >2 | >2 | 0.002 |
| MRSA | >1 | >5 | >5 | >2.5 | >5 | >5 | >5 | >5 | >5 | >5 | >2 | >2 | 0.5 |
| *B.cereus* | >1 | >1 | >1 | >1 | >1 | >1 | >1 | >1 | >1 | >1 | >2 | >2 | 0.128 |
| *S.mutans* | >2 | 2 | 0.5 | 0.5 | 0.5 | 1 | 1 | 2 | 1 | 1 | 0.0625 | 0.125 | 1 |
| *E.faecalis* | >2 | >2 | >2 | >2 | >2 | >2 | >2 | >2 | >2 | >2 | >2 | >2 | 1 |
| *E.faecium* | >2 | >2 | >2 | >2 | >2 | >2 | >2 | >2 | >2 | >2 | >2 | >2 | 0.032 |
| *B.subtilis* | >2 | >2 | >2 | >2 | >2 | >2 | >2 | >2 | >2 | >2 | >2 | >2 | <0.0625 |
| *A.baumanii* (ATCC BAA 1605) | >2 | >2 | >2 | >2 | >2 | >2 | >2 | >2 | >2 | >2 | >2 | >2 | >8 |
| *A.baumanii (Clinical C65)* | >2 | >2 | >2 | >2 | >2 | >2 | >2 | >2 | >2 | >2 | >2 | >2 | 0.0625 |
| *E. coli* | >1 | >5 | >5 | >2.5 | >5 | >5 | >5 | >5 | >5 | >5 | >2 | >2 | 0.016 |
| *P.aeruginosa* | >1 | >1 | >1 | >1 | >1 | >1 | >1 | >1 | >1 | >1 | >2 | >2 | 0.256 |
| *K.Pneumoniae* | >2 | >2 | >2 | >2 | >2 | >2 | >2 | >2 | >2 | >2 | >2 | >2 | 0.25 |
| *E.Aerogenes* | >2 | >2 | >2 | >2 | >2 | >2 | >2 | >2 | >2 | >2 | >2 | >2 | 0.5 |
| *C.albican* | >2 | >2 | >2 | >2 | >2 | >2 | >2 | >2 | >2 | >2 | 1 | 1 | 0.156 (CHX) |

CHX: Cyclohexamide; CIP*: MIC for CIP and CHX is in µg/ml.
